# Successful Blinding or Wishful Thinking? A Secondary Analysis of a Randomised Controlled Trial of Manual Therapy

**DOI:** 10.1002/ejp.70370

**Published:** 2026-09-21

**Authors:** Carlos Gevers‐Montoro, Javier Muñoz Laguna, Cesar A. Hincapié, Arantxa Ortega‐De Mues, Mathieu Piché

**Affiliations:** ^1^ Department of Psychology McGill University Montreal Quebec Canada; ^2^ Alan Edwards Centre for Research on Pain McGill University Montreal Canada; ^3^ Department of Chiropractic Université du Québec à Trois‐Rivières Trois‐Rivières Canada; ^4^ Department of Clinical Research University of Bern Bern Switzerland; ^5^ Musculoskeletal Epidemiology Research Group University of Zurich and Balgrist University Hospital Zurich Switzerland; ^6^ Epidemiology, Biostatistics and Prevention Institute (EBPI) University of Zurich Zurich Switzerland; ^7^ University Spine Centre Zurich (UWZH), Balgrist University Hospital, University of Zurich Zurich Switzerland; ^8^ Division of Epidemiology, Dalla Lana School of Public Health University of Toronto Toronto Canada; ^9^ Fujitega Research Foundation Madrid Spain; ^10^ Department of Anatomy Université du Québec à Trois‐Rivières Trois‐Rivières Canada

**Keywords:** blinding, chronic primary low back pain, masking, randomised controlled trial, spinal manipulative therapy

## Abstract

**Background:**

Blinding is rarely assessed, estimated, or reported in randomised controlled trials (RCTs). To provide contextualised understanding of participant blinding in a placebo‐controlled RCT of spinal manipulative therapy (SMT) for chronic primary low back pain (NCT05162924), we aimed to: (i) reassess blinding, (ii) explore treatment effects and adverse events by levels of beliefs, and (iii) examine whether blinding varied by treatment response.

**Methods:**

Blinding was assessed immediately after the first intervention session, at the two‐ and four‐week follow‐ups (primary endpoint). The sum Bang blinding index (BI), ranging from −2 to 2, was the primary blinding outcome for this secondary analysis. Treatment effects and adverse events were explored in blinded and unblinded participants. BIs were estimated by levels of treatment response after randomisation.

**Results:**

Of 102 randomised participants, 98 received their intervention as allocated and were included in this secondary analysis. Correct beliefs about intervention assignment in the active SMT group were balanced by incorrect beliefs in the placebo SMT group (‘wishful thinking’), suggesting adequate blinding at the study level (sum Bang BI: 0.13 [95% CI, −0.22 to 0.48]). Treatment effects were markedly larger in unblinded participants, while adverse events were comparable among blinded and unblinded participants. BIs varied by treatment response.

**Conclusion:**

Study‐level blinding of participants was adequate in this randomised placebo‐controlled trial of SMT. While treatment effects were markedly larger in unblinded participants, adverse events were comparable in blinded and unblinded participants. Blinding varied by treatment response, providing insights for the design and conduct of future trials.

**Significance Statement:**

This secondary analysis of blinding in a placebo‐controlled RCT of SMT found that blinding at the study level was adequate. Treatment effects were markedly larger in unblinded participants, highlighting the importance of blinding procedures in RCTs. Blinding findings by responder status enhance ongoing discussions regarding the feasibility of blinding in this area. Overall, insights from this secondary analysis encourage the thoughtful consideration of blinding procedures in RCTs of SMT and other physical interventions for pain.

**Abbreviations**
BI: Blinding index
CI: Confidence interval
CONSORT: CONsolidated Standards Of Reporting Trials
CPLBP: Chronic primary low back pain
NRS: Numerical rating scale
RCT: Randomised controlled trial
SD: Standard deviation
SMT: Spinal manipulative therapy
VAS: Visual analogue scale

## 1 Introduction

Low back pain is a leading cause of disability worldwide (Ferreira et al. 2023). Among low back pain phenotypes, individuals with chronic primary low back pain (CPLBP) experience pain that persists for three months or longer and cannot be attributed to any underlying condition (Nicholas et al. 2019; Nijs et al. 2024). Clinical practice guidelines for the management of CPLBP recommend and prioritise nonpharmacological interventions (World Health Organization 2023).

Spinal manipulative therapy (SMT) is a guideline‐recommended nonpharmacological intervention for CPLBP. However, the high risk of bias due to participant blinding domains remains a common methodological challenge in placebo‐controlled randomised controlled trials (RCTs) of SMT (de Zoete et al. 2026). In addition, most RCTs do not assess, estimate, or report the success of blinding (Puhl et al. 2017). To improve the interpretability of RCTs of SMT, further understanding of treatment effects and adverse events by levels of blinding, as well as blinding variation across participant characteristics and treatment response, is warranted.

Adhering to guidance to improve blinding procedures (Hohenschurz‐Schmidt et al. 2023), we conducted a placebo‐controlled RCT of SMT and assessed blinding at three timepoints after randomisation (Gevers‐Montoro et al. 2024). Our findings were reported in the original publication and suggested that blinding was successful. Yet, blinding assessment, estimation, and reporting are nuanced and may benefit from the best available statistical methods (Bang et al. 2010). Since placebo interventions aim to control for the placebo effect and minimise performance and ascertainment biases, a better understanding of participant beliefs about intervention assignment and their potential influences on trial outcomes is crucial.

Our overarching aim for this secondary analysis was to enhance the interpretability of our findings with a more comprehensive assessment, estimation, and reporting of blinding outcomes. Our specific objectives were: (i) to reassess blinding at three different timepoints after randomisation, (ii) to explore treatment effects and adverse events by levels of participant beliefs about intervention assignment at the four‐week follow‐up (primary endpoint), and (iii) to examine whether blinding varied by participant treatment responder status after randomisation.

## 2 Methods

### 2.1 Study Design and Setting

This was a secondary analysis of blinding in a two‐parallel‐group, single‐centre, placebo‐controlled RCT (Gevers‐Montoro et al. 2024). The RCT was conducted in a primary care clinic in San Lorenzo de El Escorial (Madrid, Spain). The trial was prospectively registered (NCT05162924) and approved by the institutional review board of the Fundación Jiménez Díaz Clinical Research Ethics Committee (Madrid, Spain). All participants gave written informed consent. The trial protocol and statistical analysis plan are available elsewhere (Gevers‐Montoro et al. 2023). For reporting, we adhered to the CONsolidated Standards Of Reporting Trials (CONSORT) guidelines (Hopewell et al. 2025).

### 2.2 Participants

The RCT included adults between 18 and 70 years of age with symptoms compatible with CPLBP of at least three‐month duration. Patients were excluded if they: (i) were suspected to have pain of predominant neuropathic origin, (ii) had evidence of specific pathology as the cause of their low back pain, (iii) had a diagnosis of mental illness, (iv) had pain of equal or higher intensity affecting a body region other than the low back, (v) were taking corticosteroids, opiates or anti‐cytokine medication, (vi) were pregnant, (vii) had a history of lumbar spine surgery or (viii) received chiropractic SMT in the previous 12 months. The trial protocol details the full eligibility criteria (Gevers‐Montoro et al. 2023).

### 2.3 Randomisation

Participants were randomised (1:1) using simple randomisation to active or placebo SMT intervention protocols. The allocation list was computer‐generated and concealed via a web‐based system. A researcher not involved in participant care requested randomisation and assigned participants to interventions. This researcher then communicated the assigned intervention to the intervention provider.

### 2.4 Interventions

During the informed consent process, participants were fully informed of the placebo‐controlled design and the placebo intervention procedures. They were made aware that the trial involved random allocation to one of two interventions: an active lumbar SMT or a placebo SMT closely resembling the active intervention. Participants were also informed that both intervention procedures had been previously used in clinical practice and research protocols; see the informed consent form in Data Supplement 4 of the published protocol for details (Gevers‐Montoro et al. 2023).

Participants in the active SMT group received 12 study intervention sessions over a period of four weeks. At each study session, participants received high‐velocity low‐amplitude manipulation targeting the most painful vertebral segment, bilaterally. The intervention provider performed manual palpation of the lumbosacral spine in a prone position and tailored the intervention by identifying the most painful segment. Per protocol, joint cavitations were expected and generated in the active SMT group.

Participants in the placebo SMT group also received 12 study intervention sessions over a period of four weeks. At each study session, participants received bilateral lower‐velocity thrusts to the gluteal region (Chaibi et al. 2015). The placebo SMT intervention was conceptualised to promote high placebo fidelity as it closely mimicked key structural elements of the active SMT intervention (Gevers‐Montoro et al. 2023). To foster comparability and indistinguishability with the active SMT intervention, the intervention provider performed analogous manual palpation of the lumbosacral spine in a prone position and identified the most painful segment. Per protocol, no cavitations were intentionally generated in the placebo SMT group.

A thorough description of the interventions is available in the original report (Gevers‐Montoro et al. 2024), adhering to relevant guidelines (Hoffmann et al. 2014; Hohenschurz‐Schmidt et al. 2023; Howick et al. 2020).

### 2.5 Outcomes: Blinding

Participants in the trial were assessed for blinding at three different timepoints after randomisation: immediately after the first (immediate), sixth (two‐week follow‐up), and twelfth (four‐week follow‐up) study sessions. Two blinding assessment items were used. In blinding item 1, participants were asked ‘Do you think that the treatment you have received is a real chiropractic treatment for back pain?’ with response options ‘Yes’ and ‘No’. In blinding item 2, participants were asked ‘On a numerical rating scale (NRS) of 0–100, please rate the degree of certainty for having received a real chiropractic treatment’ (0, strong certainty for having received placebo SMT; 100, strong certainty for having received active SMT; 50, uncertainty). For blinding item 2, we transformed NRS responses into five Likert‐type response categories: ≥ 75 to 100, ‘strong certainty for active SMT’; > 50 to < 75, ‘some certainty for active SMT’; 50, ‘uncertain’; > 25 to < 50, ‘some certainty for placebo SMT’; 0 to ≤ 25, ‘strong certainty for placebo SMT’.

The primary blinding outcome for this secondary analysis was blinding at the study level, as measured by the sum Bang blinding index (BI) at the four‐week follow‐up (Bang et al. 2004). The sum Bang BI is a study‐level index that measures between‐group differences in proportions of the same belief, ranging from −2 to 2. The sum Bang BI is complemented by two group‐specific and chance‐corrected indices that range from −1 (all incorrect beliefs within a group) to 1 (all correct beliefs). Group‐specific Bang BIs can be interpreted as the proportion of correct beliefs within an intervention group, beyond correct beliefs that would be expected by chance alone. A value of 0 is suggestive of ‘perfect’ blinding in both group‐ and study‐level Bang BIs, while values between −0.3 and 0.3 have been suggested to be ‘adequate’ (Muñoz Laguna, Kurmann, et al. 2025; Reid et al. 2023).

Secondary outcomes were blinding using two different indices—James BI (James et al. 1996) and Simple BI (Petroff et al. 2024). Both James and Simple BI provide a measure of study‐level blinding. The James BI measures disagreement beyond chance, returning values between 0 (complete unblinding) and 1 (complete blinding). When 50% of beliefs are correct and 50% incorrect, James BI returns 0.5. The Simple BI is a between‐group difference in probabilities of guessing active intervention. A value of 0 is suggestive of ‘perfect’ blinding for Simple BI. Additional information and comparison of available BIs is available (Muñoz Laguna, Kolahi, and Bang 2025).

To complement specific objective (i), we described blinding by levels of participant baseline characteristics. Participant characteristics were age (< 50 vs. ≥ 50 years old), sex (female vs. male), education level (high school vs. university), presence of back‐related leg pain (yes vs. no), baseline pain intensity (< 50 vs. ≥ 50 NRS), and treatment improvement expectations (≤ −70 visual analogue scale [VAS] vs. > −70 VAS, based on data distribution). The dichotomisation of continuous characteristics was ad hoc for the objectives of this secondary analysis, allowing for the exploration of blinding by key participant characteristics.

### 2.6 Outcomes: Efficacy and Safety

The primary outcome for efficacy in the trial was pain intensity at four‐week follow‐up using a NRS between 0 (no pain) and 100 (worst pain imaginable). Adverse events were reported by participants at each study session. Adverse event case report forms included data on onset, duration and participant‐reported severity. We explored differences in efficacy and safety outcomes based on blinding status.

### 2.7 Sample Size Consideration

The original trial was powered for superiority of a clinical outcome (change in pain) and aimed to recruit 50 participants per group (Gevers‐Montoro et al. 2023). Given the fixed modified intention‐to‐treat study population of 98 participants for this secondary analysis, we estimated a maximum precision of 0.32 points in the width of the 95% CI for the group‐specific Bang BI estimate for the current primary analysis (Landsman et al. 2018).

### 2.8 Statistical Analyses

We followed a modified intention‐to‐treat approach for our primary analysis and used last observation carried forward for single imputation of missing data.

For specific objective (i), BIs were reported using point estimates and 95% confidence intervals (CIs). For group‐specific Bang BIs, we estimated one‐sided CIs. For study‐level sum Bang and James BIs, we estimated two‐sided CIs. For study‐level Simple BI, CIs were based on the Wilson score method (Newcombe 1998). To complement specific objective (i), we reported BIs stratified by age, sex, education level, presence of back‐related leg pain, baseline pain intensity, and treatment expectation categories.

For specific objective (ii), we estimated conditional average treatment effects in two blinding strata at the four‐week follow‐up. The two blinding strata at the four‐week follow‐up were (i) ‘blinded’ (participants with incorrect beliefs about intervention assignment) and (ii) ‘unblinded’ (participants with correct beliefs about intervention assignment). For adverse events, we estimated differences in proportions of adverse events in two blinding strata. Since participants were blinded per protocol (Gevers‐Montoro et al. 2023), these estimands at the four‐week follow‐up were purposively selected to enhance the interpretation of primary efficacy and safety outcomes conditional on post‐randomisation blinding status (Kahan et al. 2024).

To address specific objective (iii), we explored whether blinding varied by levels of treatment response prior to each assessment and estimated BIs by responder status, both at the study and group level. Responders were participants achieving a prespecified minimal clinically important reduction in pain intensity of ≥ 25 points from baseline in the NRS (0–100) back pain intensity primary outcome (van der Roer et al. 2006).

To assess the robustness of BIs, we performed sensitivity analyses using Bayesian generalised linear mixed models with moderately informative priors. Models explored posterior probabilities of having an incorrect belief about intervention assignment (i.e., ‘blinding success’), with intervention group, pain change, and adverse events as predictors. Further details are available (see Methods S1 in Supplement).

All analyses were performed in R version 4.5.1 (R Foundation, Vienna, Austria).

## 3 Results

A total of 102 participants were randomised between 17 December 2021 and 4 July 2022. Two participants in the active SMT and two participants in the placebo SMT group did not receive the allocated intervention due to post‐randomisation exclusions, which were not influenced by intervention group assignment (Figure S1). Hence, 98 participants were included in the modified intention‐to‐treat population. The mean (SD) age was 48.3 (11.3) years, 63 (64%) were female, and 51 (52%) had completed university education. Baseline characteristics were comparable between groups (Table 1).

**TABLE 1:** Participant characteristics at baseline.

Characteristic	Active SMT (*n* = 49)	Placebo SMT (*n* = 49)
Age, mean (SD), years	48.3 (13.0)	48.3 (9.5)
Sex, No. (%)		
Female	31 (63.3)	32 (65.3)
Male	18 (36.7)	17 (34.7)
Education level, No. (%)		
High school	24 (49.0)	23 (46.9)
University	25 (51.0)	26 (53.1)
Back‐related leg pain, No. (%)	21 (42.9)	16 (32.7)
Pain intensity, mean (SD), NRS^a^	47.5 (17.3)	45.8 (19.9)
Treatment expectations, mean (SD)^b^	−68.8 (23.7)	−68.7 (27.1)

Abbreviations: No., number; NRS, numerical rating scale; SD, standard deviation.

^a^ Ranges from 0 (no pain) to 100 (worst pain imaginable).

^b^ Ranges from −100 (complete improvement expectations) to 0 (no improvement expectations).

### 3.1 Blinding

At the four‐week follow‐up, 55% of participants in the active SMT group correctly identified their assigned intervention after controlling for chance (Bang BI, 0.55 [95% CI, 0.35 to 0.75]). In the placebo SMT group, 43% of participants believed they received active SMT beyond chance levels (Bang BI, −0.43 [95% CI, −0.64 to −0.22]). Study‐level indices were comparable, suggesting adequate blinding (sum Bang BI, 0.13 [95% CI, −0.22 to 0.48]; James BI, 0.47 [95% CI, 0.38 to 0.56]; Simple BI, 0.06 [95% CI, −0.11 to 0.23]; Table 2).

**TABLE 2:** Bang, James, and Simple BI blinding outcomes.

Outcome	Active SMT	Placebo SMT	Study‐level
Timepoint	(*n* = 49)	(*n* = 49)	(95% CI)
Bang BI—blinding item 1			
Immediate	0.71 (0.55 to 0.88)	−0.63 (−0.81 to −0.45)	Sum BI, 0.08 (−0.21 to 0.37)
2 weeks	0.51 (0.31 to 0.71)	−0.55 (−0.75 to −0.35)	Sum BI, −0.04 (−0.37 to 0.29)
4 weeks	0.55 (0.35 to 0.75)	−0.43 (−0.64 to −0.22)	Sum BI, 0.13 (−0.22 to 0.48)
Bang BI—blinding item 2			
Immediate	0.31 (0.14 to 0.47)	−0.34 (−0.50 to −0.17)	Sum BI, −0.03 (−0.25 to 0.19)
2 weeks	0.32 (0.16 to 0.48)	−0.37 (−0.53 to −0.21)	Sum BI, −0.05 (−0.27 to 0.17)
4 weeks	0.32 (0.17 to 0.47)	−0.33 (−0.49 to −0.17)	Sum BI, −0.01 (−0.23 to 0.21)
James BI—blinding item 1			
Immediate			0.48 (0.41 to 0.55)
2 weeks			0.51 (0.43 to 0.59)
4 weeks			0.47 (0.38 to 0.56)
James BI—blinding item 2			
Immediate			0.63 (0.55 to 0.72)
2 weeks			0.65 (0.57 to 0.74)
4 weeks			0.62 (0.53 to 0.71)
Simple BI—blinding item 1			
Immediate			0.04 (−0.11 to 0.19)
2 weeks			−0.02 (−0.18 to 0.15)
4 weeks			0.06 (−0.11 to 0.23)
Simple BI—blinding item 2			
Immediate			−0.02 (−0.21 to 0.17)
2 weeks			−0.06 (−0.24 to 0.14)
4 weeks			−0.05 (−0.24 to 0.15)

*Note:* blinding item 1: ‘Do you think that the treatment you have received is a real chiropractic treatment for back pain?’; response options: ‘Yes’, ‘No’; blinding item 2: ‘On a numerical rating scale of 0–100, please rate the degree of certainty for having received a real chiropractic treatment’ (0, strong certainty for having received placebo SMT; 100, strong certainty for having received active SMT; 50, uncertainty); response options were transformed into five Likert‐type response categories: ≥ 75 to 100, ‘strong certainty for active SMT’; > 50 to < 75, ‘some certainty for active SMT’; 50, ‘uncertain’; > 25 to < 50, ‘some certainty for placebo SMT’; 0 to ≤ 25, ‘strong certainty for placebo SMT’; Bang BIs based on this item were weighted; Simple BIs based on this item excluded ‘uncertain’ responses.

Abbreviations: BI, blinding index; CI, confidence interval; SMT, spinal manipulative therapy.

### 3.2 Blinding Stratified by Participant Characteristics

Blinding varied by strata of participant baseline characteristics. At the four‐week follow‐up, participants ≥ 50 years old showed, on average, more incorrect beliefs about intervention assignment (< 50 years old, sum Bang BI, 0.16 [95% CI, −0.33 to 0.65]; ≥ 50 years old, sum Bang BI, 0.06 [95% CI, −0.44 to 0.56]). Compared to males, female participants also had a higher proportion of incorrect beliefs (females, sum Bang BI, −0.03 [95% CI, −0.43 to 0.49]; males, sum Bang BI, 0.36 [95% CI, −0.10 to 0.82]). More incorrect beliefs were also observed among those with high school education, rather than a university degree (high school, sum Bang BI, −0.15 [95% CI, −0.66 to 0.36]; university, sum Bang BI, 0.38 [95% CI, −0.06 to 0.82]). Compared to their stratum counterparts, more incorrect beliefs were seen among participants (i) without back‐related leg pain, (ii) with pain intensity ≥ 50 NRS and (iii) ≤ −70 VAS (greater) improvement expectations at baseline (Table S1).

### 3.3 Treatment Effects and Adverse Events by Levels of Blinding

At the four‐week follow‐up, the mean (SD) pain intensity among blinded participants was 19.5 (11.3) and 22.5 (21.1) in the active and placebo SMT groups, respectively. The conditional average treatment effect in blinded participants, expressed as a between‐group mean difference, was −3 (95% CI, −12.7 to 6.7). The mean (SD) pain intensity among unblinded participants was 15.5 (14.7) and 42.4 (23.8) in the active and placebo SMT groups. The conditional average treatment effect in unblinded participants was −26.9 (95% CI, −40.2 to −13.6). The mean between‐group differences in proportions of unique patients with adverse events were comparable among blinded (−9 percentage points [95% CI, −34 to 15]) and unblinded (3 [95% CI, −25 to 30]) participants at the four‐week follow‐up (Table 3).

**TABLE 3:** Treatment effects and adverse events.

Outcome	Active SMT	Placebo SMT	Effect (95% CI)
Blinded participants^a^	(*n* = 11)	(*n* = 35)	
Pain intensity at 4 weeks, mean (SD), NRS	19.5 (11.3)	22.5 (21.1)	−3 (−12.7 to 6.7)
Total AEs, No.	46	151	
AEs/participant ratio	4.2	4.3	
Unique participants with AEs, No. (%)	9 (81.8)	32 (91.4)	−9 (−34 to 15)^b^
Unblinded participants^c^	(*n* = 38)	(*n* = 14)	
Pain intensity at 4 weeks, mean (SD), NRS	15.5 (14.7)	42.4 (23.8)	−26.9 (−40.2 to −13.6)
Total AEs, No.	134	46	
AEs/participant ratio	3.5	3.3	
Unique participants with AEs, No. (%)	28 (73.7)	10 (71.4)	3 (−25 to 30)^b^

Abbreviations: AE, adverse event; CI, confidence interval; NRS, numerical rating scale; SD, standard deviation; SMT, spinal manipulative therapy.

^a^ ‘Blinded’ participants were those with incorrect beliefs about intervention assignment at the four‐week follow‐up.

^b^ Differences in proportions and 95% CIs were estimated without Yates' continuity correction; expressed as percentage points.

^c^ ‘Unblinded’ participants were those with correct beliefs about intervention assignment at the four‐week follow‐up.

### 3.4 Blinding by Levels of Treatment Response

**FIGURE 1:**
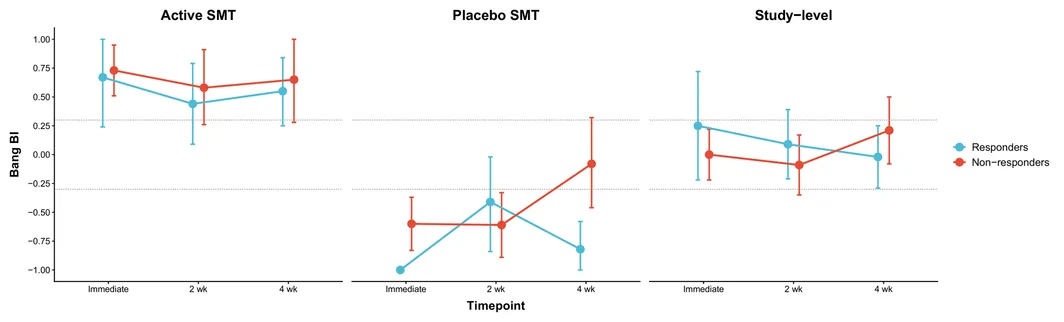
Bang BIs by responder status. BI, blinding index; SMT, spinal manipulative therapy; wk, weeks. Bang BIs approaching +1 indicate increased correct beliefs about intervention assignment beyond chance; BIs approaching −1 indicate increased incorrect beliefs beyond chance. Responders were participants achieving a prespecified minimal clinically important reduction in pain intensity of ≥ 25 points from baseline in the NRS (0–100) back pain intensity primary outcome. Dashed lines are suggested ‘adequate blinding’ limits.

At the four‐week follow‐up, participants in the active SMT group had correct beliefs about intervention assignment, irrespective of their responder status. Group‐specific Bang BIs were 0.55 (95% CI, 0.25 to 0.84) for responders and 0.65 (95% CI, 0.28 to 1.00) for non‐responders. In the placebo SMT group, beliefs about intervention assignment differed by responder status at the four‐week follow‐up. Group‐specific Bang BIs were −0.82 (95% CI, −1.00 to −0.58) for responders and −0.08 (95% CI, −0.46 to 0.32) for non‐responders. At the study level, blinding was comparable by responder status at all follow‐ups (Figure 1; Table S2).

### 3.5 Sensitivity Analyses

Bayesian sensitivity analyses yielded comparable results to the main analyses, indicating higher posterior probabilities of blinding success in the placebo SMT group, while suggesting an influence of treatment response on blinding status (Tables S3–S6 in Supplement; see also Figure S2).

## 4 Discussion

We conducted a secondary analysis of a placebo‐controlled RCT of SMT and found that study‐level blinding was adequate at the four‐week follow‐up. Participants who were older, female, had high‐school education, and without back‐related leg pain had more incorrect beliefs than their strata counterparts at the primary endpoint assessment. Treatment effects were markedly larger in unblinded participants, whereas adverse events were comparable among blinded and unblinded participants. Most participants in the active SMT group had correct beliefs about intervention assignment; those in the placebo SMT group tended to have incorrect beliefs. Responders in the placebo SMT group had more incorrect beliefs about intervention assignment than non‐responders, suggesting that blinding could be influenced by treatment response.

### 4.1 Complementing the Original RCT

The results of this secondary analysis complement those of the original report and provide contextualised understanding of blinding in the trial. Consistent with the original report (Gevers‐Montoro et al. 2024), three distinct BIs supported that blinding at the study level was successful. While blinding varied by baseline characteristics, the observed differences in treatment effects among blinded and unblinded participants are revealing. These differences highlight the importance of blinding procedures in RCTs, particularly for patient‐reported outcomes, where beliefs about intervention assignment may contribute to performance or ascertainment biases. Our comparable proportions of adverse events in blinded and unblinded participants deserve further investigation into plausible mechanisms. Our findings by responder status enhance ongoing discussions regarding the feasibility and interpretation of blinding in trials of SMT, where blinding is inherently challenging (de Zoete et al. 2026). Blinding variations across participant baseline characteristics build on previous work, providing insights into factors that may influence blinding success (Muñoz Laguna, Kolahi, and Bang 2025).

### 4.2 Further Context and Implications

Although blinding success is rarely assessed, estimated, or reported, there is previous evidence of adequate blinding at the study level in trials of SMT (Bialosky et al. 2014; Vernon et al. 2013; Walker et al. 2013), including trials using the same placebo SMT intervention protocol as the current study (Aspinall et al. 2019; Chaibi et al. 2015; Freitas et al. 2024).

Our findings may have practical implications for the methods, design, and conduct of future placebo‐controlled RCTs of SMT. For instance, extensive training and standardisation of intervention procedures may increase confidence in the delivery of placebo interventions, improving blinding at the study level (Hohenschurz‐Schmidt et al. 2023; Vernon et al. 2011). Regarding eligibility, it is important to recognise that our trial population was largely naive to the interventions as (i) receiving chiropractic SMT in the previous 12 months was an exclusion criterion and (ii) only 12.7% of participants had lifetime SMT experience (Gevers‐Montoro et al. 2024). To enhance blinding, trialists may need to balance stringent eligibility criteria (e.g., the exclusion of individuals with SMT experience) against considerations related to external validity and feasibility of trial conduct.

### 4.3 Group‐Specific Blinding

Analysis and interpretation of group‐specific BIs provided important nuances regarding the blinding scenario of our trial. Despite the considerable proportion of correct beliefs in the active group, participants in the placebo SMT group tended to believe that they received an active intervention. Results from two recent methodological trials suggest that achieving adequate group‐specific blinding in active SMT may be challenging (Muñoz Laguna, Kurmann, et al. 2024, 2025). Our ‘wishful thinking’ blinding scenario, in which both groups tended to believe they received an active treatment, may be a ‘best‐case’ scenario when attempting to minimise bias in trials of SMT (Bang 2016). Although a recent meta‐analysis does not suggest a high prevalence of ‘wishful thinking’ scenarios in trials of physical interventions for pain (Freed et al. 2021), the number of manual therapy trials contributing to the assessment, estimation, and reporting of blinding‐related data remains limited. Further methodological improvement in this area may increase our confidence in pooled blinding estimates and enhance the interpretation of blinding heterogeneity across trials.

### 4.4 Blinding and Treatment Response

Our findings revealed large differences in follow‐up pain intensity between unblinded participants in the active and placebo SMT groups. Mean pain ratings were comparable among those in the active SMT group, irrespective of their blinding status. However, on average, unblinded participants receiving placebo SMT rated their pain almost 20 points higher than their blinded counterpart. This finding suggests that participant beliefs about intervention assignment may have influenced their patient‐reported pain outcome assessments, which could be consistent with large nonspecific effects attributed to manual therapy controls (Hohenschurz‐Schmidt et al. 2024). In addition to these considerations, our findings highlight that responders within the placebo SMT group were strongly biased towards having incorrect beliefs about intervention assignment, whereas non‐responders displayed beliefs compatible with chance. Treatment effects may have influenced participant beliefs about intervention assignment in the placebo SMT group; however, temporality cannot be elucidated. Although a meta‐epidemiological study has suggested that blinding may be less important than often believed (Moustgaard et al. 2020), our findings support a more nuanced interpretation of the interplay between blinding and treatment effects.

It should be noted that treatment responses in the control group may not be explained by intervention assignment beliefs and placebo effects alone. Other nonspecific factors related to the therapeutic and trial context may contribute to the observed outcomes (Sean et al. 2024). Future RCTs may consider measuring and controlling for factors related to the intervention provider‐participant interactions to improve the interpretability of treatment effects.

### 4.5 Strengths and Limitations

Our study has strengths. First, it is based on an RCT with comparable groups at baseline, high adherence to intervention protocols, appropriate analysis to estimate the effect of randomised assignment to intervention, and available data for nearly all participants at the primary endpoint assessment. Second, participant beliefs about intervention assignment were assessed at three different timepoints after randomisation, which allowed us to estimate the success of blinding over a four‐week follow‐up period. Third, we ensured comprehensiveness in blinding assessment and estimation by using two different blinding assessment items and three available indices.

Our study also has limitations. First, we conducted an exploratory secondary analysis of blinding that was not prespecified in the published protocol. Hence, this analysis was not powered to detect differences in subgroups. Second, our blinding estimates are restricted to the primary endpoint assessment at four weeks. Maintenance of blinding beyond this timepoint is uncertain. Third, to address our second and third objectives, we stratified by levels of post‐randomisation variables, which may have introduced selection bias. Fourth, interventions in this trial were delivered by a single provider in both groups, which may compromise the external validity of our findings.

## 5 Conclusion

Blinding of participants at the study level was adequate in this placebo‐controlled RCT of SMT, although it varied by group assignment and participant characteristics. While treatment effects were markedly larger in unblinded participants, prior treatment response may have influenced beliefs about intervention assignment in the placebo group. Our results encourage thoughtful consideration of blinding in RCTs of SMT, including the comprehensive and transparent reporting of blinding estimates, the exploration of blinding variation in patient subgroups, and the examination of treatment effects and adverse events by levels of participant beliefs.

## Author Contributions

Carlos Gevers‐Montoro and Javier Muñoz Laguna contributed equally to this secondary analysis and are co‐first authors. Carlos Gevers‐Montoro contributed to conceptualisation, data collection, curation and analysis, and writing; Javier Muñoz Laguna contributed to conceptualisation, methodology, data curation and analysis, and writing; Cesar A. Hincapié was involved in writing and supervision; Arantxa Ortega‐De Mues contributed to data collection, writing, and supervision; Mathieu Piché was responsible for conceptualisation, data collection, writing, supervision, and funding acquisition.

## Supporting information


**Figure S1:** Participant recruitment, randomisation, and follow‐up.
**Table S1:** Bang BIs stratified by levels of participant baseline characteristics.
**Table S2:** Bang BIs stratified by levels of participant response.
**Methods S1:** Bayesian sensitivity analysis.
**Results S1:** Bayesian sensitivity analysis.
**Table S3:** Model coefficients from Bayesian generalised linear mixed models.
**Table S4:** Posterior probabilities of blinding success by intervention group.
**Table S5:** Effects of pain changes and adverse events on blinding success.
**Table S6:** Blinding success over time by intervention group.
**Figure S2:** Bayesian summary panel plot.

## References

[ejp70370-bib-0001] Aspinall, S. L. , A. Jacques , C. Leboeuf‐Yde , S. J. Etherington , and B. F. Walker . 2019. “No Difference in Pressure Pain Threshold and Temporal Summation After Lumbar Spinal Manipulation Compared to Sham: A Randomised Controlled Trial in Adults With Low Back Pain.” Musculoskeletal Science & Practice 43: 18–25. 10.1016/j.msksp.2019.05.011 31176287

[ejp70370-bib-0002] Bang, H. 2016. “Random Guess and Wishful Thinking Are the Best Blinding Scenarios.” Contemporary Clinical Trials Communications 3: 117–121. 10.1016/j.conctc.2016.05.003 PMC5096460 27822568

[ejp70370-bib-0003] Bang, H. , S. P. Flaherty , J. Kolahi , and J. Park . 2010. “Blinding Assessment in Clinical Trials: A Review of Statistical Methods and a Proposal of Blinding Assessment Protocol.” Clinical Research and Regulatory Affairs 27: 42–51.

[ejp70370-bib-0004] Bang, H. , L. Ni , and C. E. Davis . 2004. “Assessment of Blinding in Clinical Trials.” Controlled Clinical Trials 25: 143–156. 10.1016/j.cct.2003.10.016 15020033

[ejp70370-bib-0005] Bialosky, J. E. , S. Z. George , M. E. Horn , D. D. Price , R. Staud , and M. E. Robinson . 2014. “Spinal Manipulative Therapy‐Specific Changes in Pain Sensitivity in Individuals With Low Back Pain (NCT01168999).” Journal of Pain 15: 136–148. 10.1016/j.jpain.2013.10.005 PMC3946602 24361109

[ejp70370-bib-0006] Chaibi, A. , J. Šaltytė Benth , and M. Bjørn Russell . 2015. “Validation of Placebo in a Manual Therapy Randomized Controlled Trial.” Scientific Reports 5: 11774. 10.1038/srep11774 PMC4491841 26145718

[ejp70370-bib-0007] de Zoete, A. , T. Innocenti , M. J. Petrozzi , et al. 2026. “Spinal Manipulative Therapy for Adults With Chronic Low Back Pain.” Cochrane Database of Systematic Reviews 1: CD008112. 10.1002/14651858.CD008112.pub3 PMC12774432 41494147

[ejp70370-bib-0008] Ferreira, M. L. , K. de Luca , L. M. Haile , et al. 2023. “Global, Regional, and National Burden of Low Back Pain, 1990–2020, Its Attributable Risk Factors, and Projections to 2050: A Systematic Analysis of the Global Burden of Disease Study 2021.” Lancet Rheumatology 5: e316–e329. 10.1016/S2665-9913(23)00098-X PMC10234592 37273833

[ejp70370-bib-0009] Freed, B. , B. Williams , X. Situ , et al. 2021. “Blinding, Sham, and Treatment Effects in Randomized Controlled Trials for Back Pain in 2000–2019: A Review and Meta‐Analytic Approach.” Clinical Trials 18: 361–370. 10.1177/1740774520984870 PMC8172416 33478258

[ejp70370-bib-0010] Freitas, J. P. , L. A. Corrêa , J. V. Bittencourt , K. M. Armstrong , N. Meziat‐Filho , and L. A. C. Nogueira . 2024. “One Spinal Manipulation Session Reduces Local Pain Sensitivity but Does Not Affect Postural Stability in Individuals With Chronic Low Back Pain: A Randomised, Placebo‐Controlled Trial.” Chiropractic & Manual Therapies 32: 1–12. 10.1186/s12998-024-00541-4 PMC11143588 38822395

[ejp70370-bib-0011] Gevers‐Montoro, C. , A. Ortega‐De Mues , and M. Piché . 2023. “Mechanisms of Chiropractic Spinal Manipulative Therapy for Patients With Chronic Primary Low Back Pain: Protocol for a Mechanistic Randomised Placebo‐Controlled Trial.” BMJ Open 13: e065999. 10.1136/bmjopen-2022-065999 PMC9923302 36764718

[ejp70370-bib-0012] Gevers‐Montoro, C. , B. Romero‐Santiago , I. Medina‐García , et al. 2024. “Reduction of Chronic Primary Low Back Pain by Spinal Manipulative Therapy Is Accompanied by Decreases in Segmental Mechanical Hyperalgesia and Pain Catastrophizing: A Randomized Placebo‐Controlled Dual‐Blind Mixed Experimental Trial.” Journal of Pain 25: 104500. 10.1016/j.jpain.2024.02.014 38369221

[ejp70370-bib-0013] Hoffmann, T. C. , P. P. Glasziou , I. Boutron , et al. 2014. “Better Reporting of Interventions: Template for Intervention Description and Replication (TIDieR) Checklist and Guide.” BMJ 348: g1687. 10.1136/bmj.g1687 24609605

[ejp70370-bib-0014] Hohenschurz‐Schmidt, D. , J. Phalip , J. Chan , et al. 2024. “Placebo Analgesia in Physical and Psychological Interventions: Systematic Review and Meta‐Analysis of Three‐Armed Trials.” European Journal of Pain 28: 513–531. 10.1002/ejp.2205 37985188

[ejp70370-bib-0015] Hohenschurz‐Schmidt, D. , L. Vase , W. Scott , et al. 2023. “Recommendations for the Development, Implementation, and Reporting of Control Interventions in Efficacy and Mechanistic Trials of Physical, Psychological, and Self‐Management Therapies: The CoPPS Statement.” BMJ 381: e072108. 10.1136/bmj-2022-072108 37230508

[ejp70370-bib-0016] Hopewell, S. , A.‐W. Chan , G. S. Collins , et al. 2025. “CONSORT 2025 Statement: Updated Guideline for Reporting Randomised Trials.” BMJ 389: e081123. 10.1136/bmj-2024-081123 PMC11995449 40228833

[ejp70370-bib-0017] Howick, J. , R. K. Webster , J. L. Rees , et al. 2020. “TIDieR‐Placebo: A Guide and Checklist for Reporting Placebo and Sham Controls.” PLoS Medicine 17: e1003294. 10.1371/journal.pmed.1003294 PMC7505446 32956344

[ejp70370-bib-0018] James, K. E. , D. A. Bloch , K. K. Lee , H. C. Kraemer , and R. K. Fuller . 1996. “An Index for Assessing Blindness in a Multi‐Centre Clinical Trial: Disulfiram for Alcohol Cessation–a VA Cooperative Study.” Statistics in Medicine 15: 1421–1434. 10.1002/(SICI)1097-0258(19960715)15:13<1421::AID-SIM266>3.0.CO;2-H 8841652

[ejp70370-bib-0019] Kahan, B. C. , J. Hindley , M. Edwards , S. Cro , and T. P. Morris . 2024. “The Estimands Framework: A Primer on the ICH E9(R1) Addendum.” BMJ 384: e076316. 10.1136/bmj-2023-076316 PMC10802140 38262663

[ejp70370-bib-0020] Landsman, V. , M. Fillery , H. Vernon , and H. Bang . 2018. “Sample Size Calculations for Blinding Assessment.” Journal of Biopharmaceutical Statistics 28: 857–869. 10.1080/10543406.2017.1399898 PMC5960641 29157126

[ejp70370-bib-0021] Moustgaard, H. , G. L. Clayton , H. E. Jones , et al. 2020. “Impact of Blinding on Estimated Treatment Effects in Randomised Clinical Trials: Meta‐Epidemiological Study.” BMJ 368: l6802. 10.1136/bmj.l6802 PMC7190062 31964641

[ejp70370-bib-0022] Muñoz Laguna, J. , J. Kolahi , and H. Bang . 2025. “Unmasking Three Blinding Indices for Randomized Controlled Trials: Comparison and Application.” Contemporary Clinical Trials Communications 48: 101553. 10.1016/j.conctc.2025.101553 PMC12547027 41142043

[ejp70370-bib-0023] Muñoz Laguna, J. , A. Kurmann , L. Hofstetter , et al. 2025. “‘Which Treatment Do You Believe You Received?’ A Randomised Blinding Feasibility Trial of Spinal Manual Therapy.” Chiropractic & Manual Therapies 33: 4. 10.1186/s12998-024-00561-0 PMC11730787 39810207

[ejp70370-bib-0024] Muñoz Laguna, J. , E. Nyantakyi , U. Bhattacharyya , et al. 2024. “Is Blinding in Studies of Manual Soft Tissue Mobilisation of the Back Possible? A Feasibility Randomised Controlled Trial With Swiss Graduate Students.” Chiropractic & Manual Therapies 32: 3. 10.1186/s12998-023-00524-x PMC10826218 38287417

[ejp70370-bib-0025] Newcombe, R. G. 1998. “Interval Estimation for the Difference Between Independent Proportions: Comparison of Eleven Methods.” Statistics in Medicine 17: 873–890. 10.1002/(sici)1097-0258(19980430)17:8<873::aid-sim779>3.0.co;2-i 9595617

[ejp70370-bib-0026] Nicholas, M. , J. W. S. Vlaeyen , W. Rief , et al. 2019. “The IASP Classification of Chronic Pain for ICD‐11: Chronic Primary Pain.” Pain 160: 28–37. 10.1097/j.pain.0000000000001390 30586068

[ejp70370-bib-0027] Nijs, J. , E. Kosek , A. Chiarotto , et al. 2024. “Nociceptive, Neuropathic, or Nociplastic Low Back Pain? The Low Back Pain Phenotyping (BACPAP) Consortium's International and Multidisciplinary Consensus Recommendations.” Lancet Rheumatology 6: e178–e188. 10.1016/S2665-9913(23)00324-7 38310923

[ejp70370-bib-0028] Petroff, D. , M. Bacak , N. Dagres , P. Dilk , and R. Wachter . 2024. “A Simple Blinding Index for Randomized Controlled Trials.” Contemporary Clinical Trials Communications 42: 101393. 10.1016/j.conctc.2024.101393 PMC11647154 39686958

[ejp70370-bib-0029] Puhl, A. A. , C. J. Reinhart , J. B. Doan , and H. Vernon . 2017. “The Quality of Placebos Used in Randomized, Controlled Trials of Lumbar and Pelvic Joint Thrust Manipulation—A Systematic Review.” Spine Journal 17: 445–456. 10.1016/j.spinee.2016.11.003 27888138

[ejp70370-bib-0030] Reid, E. , O. F. Kamlin , F. Orsini , et al. 2023. “Success of Blinding a Procedural Intervention in a Randomised Controlled Trial in Preterm Infants Receiving Respiratory Support.” Clinical Trials 20: 479–485. 10.1177/17407745231171647 37144610

[ejp70370-bib-0031] Sean, M. , A. Coulombe‐Lévêque , W. Nadeau , et al. 2024. “Counting Your Chickens Before They Hatch: Improvements in an Untreated Chronic Pain Population, Beyond Regression to the Mean and the Placebo Effect.” Pain Reports 9: e1157. 10.1097/PR9.0000000000001157 PMC11057814 38689593

[ejp70370-bib-0032] van der Roer, N. , R. W. J. G. Ostelo , G. E. Bekkering , M. W. van Tulder , and H. C. W. de Vet . 2006. “Minimal Clinically Important Change for Pain Intensity, Functional Status, and General Health Status in Patients With Nonspecific Low Back Pain.” Spine 31: 578–582. 10.1097/01.brs.0000201293.57439.47 16508555

[ejp70370-bib-0033] Vernon, H. , A. Puhl , and C. Reinhart . 2011. “Systematic Review of Clinical Trials of Cervical Manipulation: Control Group Procedures and Pain Outcomes.” Chiropractic & Manual Therapies 19: 3. 10.1186/2045-709X-19-3 PMC3039829 21247413

[ejp70370-bib-0034] Vernon, H. , J. T. Triano , D. Soave , M. Dinulos , K. Ross , and S. Tran . 2013. “Retention of Blinding at Follow‐Up in a Randomized Clinical Study Using a Sham‐Control Cervical Manipulation Procedure for Neck Pain: Secondary Analyses From a Randomized Clinical Study.” Journal of Manipulative and Physiological Therapeutics 36: 522–526. 10.1016/j.jmpt.2013.06.005 24011656

[ejp70370-bib-0035] Walker, B. F. , J. J. Hebert , N. J. Stomski , B. Losco , and S. D. French . 2013. “Short‐Term Usual Chiropractic Care for Spinal Pain: A Randomized Controlled Trial.” Spine 38: 2071–2078. 10.1097/01.brs.0000435032.73187.c7 24026159

[ejp70370-bib-0036] World Health Organization . 2023. “WHO Guideline for Non‐Surgical Management of Chronic Primary Low Back Pain in Adults in Primary and Community Care Settings.” 38198579

