# Supplementary material for: Successful Blinding or Wishful Thinking? A Secondary Analysis of a Randomised Controlled Trial of Manual Therapy
Source: Eur J Pain. 2026 Sep 21;30(8):e70370. doi: 10.1002/ejp.70370 (PMC13591290; doi:10.1002/ejp.70370)
Supplement: Supplementary file 1 [file EJP-30-0-s001.docx]

**Supplement**

Gevers-Montoro, C., Muñoz Laguna, J., Hincapié, C.A., Ortega-De Mues, A., Piché, M. 2026. “Successful Blinding or Wishful Thinking? A Secondary Analysis of a Randomised Controlled Trial of Manual Therapy”. *Eur J Pain*.

**Figure S1.** Participant recruitment, randomisation, and follow-up	2

**Table S1.** Bang BIs stratified by levels of participant baseline characteristics	3

**Table S2.** Bang BIs stratified by levels of participant response	6

**Methods S1.** Bayesian sensitivity analysis	7

**Results S1.** Bayesian sensitivity analysis	9

**Table S3.** Model coefficients from Bayesian generalised linear mixed models	9

**Table S4.** Posterior probabilities of blinding success by intervention group	10

**Table S5.** Effects of pain changes and adverse events on blinding success	10

**Table S6.** Blinding success over time by intervention group	11

**Figure S2.** Bayesian summary panel plot.	12

**References S1**	12

# **Figure S1.** Participant recruitment, randomisation, and follow-up

# **Table S1.** Bang BIs stratified by levels of participant baseline characteristics

| **Characteristic**  **Timepoint** | **Active SMT** | **Placebo SMT** | **Study-level**  **(95% CI)** |
| --- | --- | --- | --- |
| *Age* |  |  |  |
| *<50 years old* | (n = 25) | (n = 28) |  |
| Immediate | 0.60 (0.34 to 0.86) | -0.50 (-0.77 to -0.23) | Sum BI, 0.10 (-0.34 to 0.54) |
| 2 wk | 0.60 (0.34 to 0.86) | -0.50 (-0.77 to -0.23) | Sum BI, 0.10 (-0.34 to 0.54) |
| 4 wk | 0.52 (0.24 to 0.80) | -0.36 (-0.65 to -0.07) | Sum BI, 0.16 (-0.33 to 0.65) |
| *≥50 years old* | (n = 24) | (n = 21) |  |
| Immediate | 0.83 (0.65 to 1.02) | -0.81 (-1.02 to -0.60) | Sum BI, 0.02 (-0.31 to 0.35) |
| 2 wk | 0.42 (0.11 to 0.72) | -0.62 (-0.90 to -0.34) | Sum BI, -0.20 (-0.70 to 0.30) |
| 4 wk | 0.58 (0.31 to 0.86) | -0.52 (-0.83 to -0.22) | Sum BI, 0.06 (-0.44 to 0.56) |
| *Sex* |  |  |  |
| *Female* | (n = 31) | (n = 32) |  |
| Immediate | 0.61 (0.38 to 0.85) | -0.75 (-0.94 to -0.56) | Sum BI, -0.14 (-0.50 to 0.22) |
| 2 wk | 0.29 (0.01 to 0.57) | -0.56 (-0.80 to -0.32) | Sum BI, -0.27 (-0.71 to 0.17) |
| 4 wk | 0.35 (0.08 to 0.63) | -0.38 (-0.64 to -0.11) | Sum BI, -0.03 (-0.43 to 0.49) |
| *Male* | (n = 18) | (n = 17) |  |
| Immediate | 0.89 (0.71 to 1.07) | -0.41 (-0.78 to -0.05) | Sum BI, 0.48 (0.00 to 0.96) |
| 2 wk | 0.89 (0.71 to 1.07) | -0.53 (-0.87 to -0.19) | Sum BI, 0.36 (-0.10 to 0.82) |
| 4 wk | 0.89 (0.71 to 1.07) | -0.53 (-0.87 to -0.19) | Sum BI, 0.36 (-0.10 to 0.82) |
| *Education* |  |  |  |
| *High school* | (n = 24) | (n = 23) |  |
| Immediate | 0.58 (0.31 to 0.86) | -0.57 (-0.85 to -0.28) | Sum BI, 0.01 (-0.46 to 0.48) |
| 2 wk | 0.25 (-0.08 to 0.58) | -0.48 (-0.78 to -0.18) | Sum BI, -0.23 (-0.76 to 0.30) |
| 4 wk | 0.33 (0.02 to 0.65) | -0.48 (-0.78 to -0.18) | Sum BI, -0.15 (-0.66 to 0.36) |
| *University* | (n = 25) | (n = 26) |  |
| Immediate | 0.84 (0.66 to 1.02) | -0.69 (-0.93 to -0.46) | Sum BI, 0.15 (-0.20 to 0.50) |
| 2 wk | 0.76 (0.55 to 0.97) | -0.62 (-0.87 to -0.36) | Sum BI, 0.14 (-0.25 to 0.53) |
| 4 wk | 0.76 (0.55 to 0.97) | -0.38 (-0.68 to -0.09) | Sum BI, 0.38 (-0.06 to 0.82) |
| *Back-related leg pain* |  |  |  |
| *Present* | (n = 21) | (n = 16) |  |
| Immediate | 0.71 (0.46 to 0.97) | -0.38 (-0.76 to 0.01) | Sum BI, 0.33 (-0.21 to 0.87) |
| 2 wk | 0.52 (0.22 to 0.83) | -0.25 (-0.65 to 0.15) | Sum BI, 0.27 (-0.33 to 0.87) |
| 4 wk | 0.62 (0.34 to 0.90) | 0.00 (-0.41 to 0.41) | Sum BI, 0.62 (0.03 to 1.21) |
| *Absent* | (n = 28) | (n = 33) |  |
| Immediate | 0.71 (0.50 to 0.93) | -0.76 (-0.94 to -0.57) | Sum BI, -0.05 (-0.38 to 0.28) |
| 2 wk | 0.50 (0.23 to 0.77) | -0.70 (-0.90 to -0.49) | Sum BI, -0.20 (-0.59 to 0.19) |
| 4 wk | 0.50 (0.23 to 0.77) | -0.64 (-0.86 to -0.42) | Sum BI, -0.14 (-0.54 to 0.26) |
| *Pain intensity* |  |  |  |
| *<50 NRS* | (n = 20) | (n = 23) |  |
| Immediate | 0.80 (0.58 to 1.02) | -0.74 (-0.97 to -0.51) | Sum BI, 0.06 (-0.31 to 0.43) |
| 2 wk | 0.60 (0.31 to 0.89) | -0.74 (-0.97 to -0.51) | Sum BI, -0.14 (-0.59 to 0.31) |
| 4 wk | 0.80 (0.58 to 1.02) | -0.48 (-0.78 to -0.18) | Sum BI, 0.32 (-0.12 to 0.76) |
| *≥50 NRS* | (n = 29) | (n = 26) |  |
| Immediate | 0.65 (0.42 to 0.89) | -0.54 (-0.81 to -0.27) | Sum BI, 0.11 (-0.32 to 0.54) |
| 2 wk | 0.45 (0.18 to 0.72) | -0.38 (-0.68 to -0.09) | Sum BI, 0.07 (-0.42 to 0.56) |
| 4 wk | 0.38 (0.10 to 0.66) | -0.38 (-0.68 to -0.09) | Sum BI, 0.00 (-0.49 to 0.49) |
| *Expectations for improvement* |  |  |  |
| *>-70 VAS (lower)* | (n = 23) | (n = 20) |  |
| Immediate | 0.65 (0.39 to 0.91) | -0.70 (-0.96 to -0.44) | Sum BI, -0.05 (-0.49 to 0.39) |
| 2 wk | 0.57 (0.28 to 0.85) | -0.50 (-0.82 to -0.18) | Sum BI, 0.07 (-0.43 to 0.57) |
| 4 wk | 0.48 (0.18 to 0.78) | -0.20 (-0.56 to 0.16) | Sum BI, 0.28 (-0.28 to 0.84) |
| *≤-70 VAS (higher)* | (n = 26) | (n = 29) |  |
| Immediate | 0.77 (0.56 to 0.98) | -0.59 (-0.83 to -0.34) | Sum BI, 0.18 (-0.21 to 0.57) |
| 2 wk | 0.46 (0.18 to 0.75) | -0.59 (-0.83 to -0.34) | Sum BI, -0.13 (-0.57 to 0.31) |
| 4 wk | 0.62 (0.36 to 0.87) | -0.59 (-0.83 to -0.34) | Sum BI, 0.03 (-0.38 to 0.45) |
| Abbreviations: BI, blinding index; CI, confidence interval; NRS, Numerical Rating Scale; SMT, spinal manipulative therapy; VAS, Visual Analogue Scale; wk, weeks. | | | |

# **Table S2.** Bang BIs stratified by levels of participant response

| **Characteristic**  **Timepoint** | **Active SMT** | **Placebo SMT** | **Study-level**  **(95% CI)** |
| --- | --- | --- | --- |
| ***Responders^a^*** |  |  |  |
| Immediate | (n = 12) | (n = 4) | (n = 16) |
|  | 0.67 (0.24 to 1.00) | -1.00 (-1.00 to -1.00) | Sum BI, 0.25 (-0.22 to 0.72) |
| 2 wk | (n = 25) | (n = 17) | (n = 42) |
|  | 0.44 (0.09 to 0.79) | -0.41 (-0.84 to -0.02) | Sum BI, 0.09 (-0.21 to 0.39) |
| 4 wk | (n = 31) | (n = 22) | (n = 53) |
|  | 0.55 (0.25 to 0.84) | -0.82 (-1.00 to -0.58) | Sum BI, -0.02 (-0.29 to 0.25) |
| ***Non-responders*** |  |  |  |
| Immediate | (n = 37) | (n = 45) | (n = 82) |
|  | 0.73 (0.51 to 0.95) | -0.60 (-0.83 to -0.37) | Sum BI, 0.00 (-0.22 to 0.22) |
| 2 wk | (n = 24) | (n = 31) | (n = 55) |
|  | 0.58 (0.26 to 0.91) | -0.61 (-0.89 to -0.33) | Sum BI, -0.09 (-0.35 to 0.17) |
| 4 wk | (n = 17) | (n = 26) | (n = 43) |
|  | 0.65 (0.28 to 1.00) | -0.08 (-0.46 to 0.32) | Sum BI, 0.21 (-0.08 to 0.50) |
| Abbreviations: BI, blinding index; CI, confidence interval; SMT, spinal manipulative therapy; wk, weeks.  ^a^ Responders were participants achieving a prespecified minimal clinically important reduction in pain intensity of ≥25 points from baseline in the primary back pain intensity outcome, NRS (0–100). | | | |

# **Methods S1.** Bayesian sensitivity analysis

We conducted a sensitivity analysis with the aim to understand whether patterns in symptom changes or in the number of adverse events could be influencing individual beliefs about intervention assignment at the three timepoints. We also aimed to explore whether these effects differed by group. We built Bayesian generalised linear mixed models (GLMM) with a Bernoulli likelihood (logit) to predict the probability of successful blinding (Gelman et al., 1995; Gelman and Hill 2007; Spiegelhalter et al., 2004). The model used blinding item 1 as dependent variable. For participants in the active SMT group, guessing ‘No’ in response to blinding item 1 was defined as ‘blinding success’, whereas for the placebo group, guessing ‘Yes’ defined success. Hence, the outcome variable ‘blinding success’ was binary-coded (1 = success, 0 = failure). Based on data suggesting that higher rates of adverse events and larger treatment effect sizes may influence blinding (Colagiuri et al., 2019), pain slopes and the cumulative number of adverse events reported were computed for the periods preceding each blinding assessment and entered as standardised z-scores in the model. Timepoints (immediate, 2 wk, 4 wk) and group were entered as factors. The model included all main effects (timepoint, pain change, adverse events, and treatment group) and their interactions, with random intercepts and random slopes of time within participants to account for repeated measures (Gelman and Hill 2007).

We used moderately informative priors, which improve estimation stability in hierarchical models (Gelman et al., 2008). Specifically, we used Normal(0, 1.5; mean and SD on the log-odds scale) priors for fixed effects, which assumes no effect with substantial deviations, consistent with recommendations to encode weakly informative domain knowledge (Gelman et al., 2017). For group contrasts, we used Normal(−0.4, 1.0) to reflect expectations of higher success in the placebo group. The intercept and random-effect standard deviations (SDs) were given Student-t(3, 0, 2.0) priors, and correlations among random effects were regularised with a Lewandowski–Kurowicka–Joe (LKJ) prior with shape parameter 2 (Lewandowski et al., 2009). Analyses were performed in *R* (v4.3.0) using the *brms* (v2.20.0) package (Bürkner 2017), with *Stan* (v2.32.0) (Carpenter et al., 2017) as backend for Hamiltonian Monte Carlo (HMC) sampling for Bayesian inference (Neal 2011). The model was specified as a hierarchical logistic regression with random intercepts for participants and timepoints to account for within-subject clustering.

The model was fitted using 4 parallel chains with 3000 iterations each (1500 warmup and 1500 post-warmup), yielding 6000 post-warmup samples for inference. Conservative settings were used for stable sampling (*adapt_delta* = 0.99; *max_treedepth* = 15) to ensure convergence (Betancourt 2019). Model convergence was assessed with the potential scale reduction factor (R-hat) and effective sample size (ESS) (Vehtari et al., 2021). The posterior distributions of the model parameters were then analysed to estimate the probability of successful blinding across groups and timepoints, and to quantify the uncertainty around these estimates.

# **Results S1.** Bayesian sensitivity analysis

We analysed 291 blinding assessments from a total of 98 participants across three timepoints (see **Figure S2**). The GLMM showed good convergence (all R-hat ~ 1.00; bulk and tail ESS > 1000; see **Table S3**). The model revealed a main effect of treatment group on the likelihood of blinding success. In the placebo group, the posterior median probability of blinding success was 0.95 (95% credible interval [CI]: 83.9–99.5%), compared to only 0.06 (95% CI: 0.4–18.0%) in the active SMT group. The fixed effect for the head-to-head group comparison (active vs. placebo) was strongly negative (log-odds = –5.63, 95% CI: –7.85 to –3.82), suggesting that achieving blinding was much more likely for participants on the placebo group (**Tables S3, S4**, and **S5**).

## **Table S3.** Model coefficients from Bayesian generalised linear mixed models

| **Parameter** | **Posterior Mean** | **95% CI** |
| --- | --- | --- |
| ***Main effects*** |  |  |
| Timepoint: Immediate (vs. 4 wk) | -0.06 | [-1.29, 1.17] |
| Timepoint: 2 wk (vs. 4 wk) | -0.37 | [-1.46, 0.70] |
| Pain Slope (z-score) | -1.14 | [-2.79, 0.47] |
| Adverse Events (z-score) | 0.08 | [-1.08, 1.22] |
| Group: Active SMT (vs. Placebo SMT) | -5.63 | [-7.85, -3.82] |
| ***Interactions*** |  |  |
| Pain Slope × Immediate | 0.25 | [-1.34, 1.85] |
| Pain Slope × 2 wk | 1.75 | [-0.58, 4.09] |
| Adverse Events × Immediate | 0.21 | [-1.38, 1.80] |
| Adverse Events × 2 wk | -0.21 | [-1.15, 0.72] |
| Pain Slope × Active SMT | 1.14 | [0.05, 2.36] |
| Adverse Events × Active SMT | 0.54 | [-0.60, 1.74] |
| *‘CI’ = credible intervals; ‘wk’ = weeks; ‘SMT’ = spinal manipulative therapy. Coefficients are on the log-odds scale. Positive values increase the probability of blinding success. Reference categories: Placebo SMT group, 4 wk timepoint. All continuous predictors are standardised (z-scores). All R-hat were ~ 1.* | | |

## **Table S4.** Posterior probabilities of blinding success by intervention group

| **Group** | **Posterior Mean** | **95% CI** | **Odds Ratio** | **OR 95% CI** |
| --- | --- | --- | --- | --- |
| Placebo SMT | 946 | 0.84, 0.99 | 1.00 | – |
| Active SMT | 62 | 0.004, 0.18 | 0.004 | 0.001, 0.06 |
| *‘OR’ = odds ratio; ‘CI’ = credible intervals; ‘wk’ = weeks; ‘SMT’ = spinal manipulative therapy. Results from Bayesian generalised linear mixed model with Bernoulli likelihood and logit link. Posterior probabilities represent the probability of successful blinding (binary Yes/No responses) averaged across timepoints. Odds ratios compare SMT to Placebo group.* | | | | |

Across groups and timepoints, increases in pain intensity (worsening symptoms) showed a trend towards reducing blinding success. Posterior median probability of blinding success was 0.65 at –1SD, 0.52 at the mean, and 0.37 at +1SD in standardised pain slopes (**Table S5**). The main effect of pain slope was negative, although with wide credible intervals (log-odds = –1.14, 95% CI: –2.79 to 0.47, **Table S3**). The interaction between pain slope and group was positive, suggesting that pain changes influenced blinding success differently in each group (log-odds = 1.14, 95% CI: 0.05–2.36; **Table S3**). On the other hand, the main effect estimate of adverse events (log-odds = 0.08, 95% CI: –1.08 to 1.22), and the interaction by group (log-odds = 0.54, 95% CI: –0.60 to 1.74; **Table S3**) approached zero.

## **Table S5.** Effects of pain changes and adverse events on blinding success

| **Clinical outcomes** | **Posterior Mean** | **95% CI** |
| --- | --- | --- |
| ***Pain changes*** |  |  |
| Improvement (-1 SD) | 0.65 | 0.20, 0.97 |
| No Change (0 SD) | 0.52 | 0.24, 0.76 |
| Worsening (+1 SD) | 0.37 | 0.13, 0.70 |
| ***Adverse events*** |  |  |
| Low AEs (-1 SD) | 0.43 | 0.16, 0.72 |
| Average AEs (0 SD) | 0.52 | 0.24, 0.76 |
| High AEs (+1 SD) | 0.60 | 0.23, 0.93 |
| *‘CI’ = credible intervals; ‘SD’ = standard deviation; ‘AEs’ = adverse events. Results averaged across treatment groups and timepoints. Pain changes and adverse events are standardised (z-scores). Posterior probabilities represent the probability of successful blinding at different levels of pain change and adverse event frequency.* | | |

Blinding success was stable across timepoints (**Table S6**). Success rates were consistently high in the placebo group (0.94 at immediate, 0.92 at 2 wk, and 0.96 at 4 wk), and consistently low for the active SMT group (0.06 at immediate, 0.04 at 2 wk, and 0.09 at 4 wk), without credible evidence for an effect of timepoint, or for its interaction with pain slopes or AEs (log-odds approaching zero, see **Table S3**).

## **Table S6.** Blinding success over time by intervention group

| **Timepoint** | **Group** | **Posterior Mean** | **95% CI** |
| --- | --- | --- | --- |
| Immediate | Placebo SMT | 0.94 | 0.75, 0.99 |
| 2 wk | Placebo SMT | 0.92 | 0.75, 0.99 |
| 4 wk | Placebo SMT | 0.96 | 0.84, 0.99 |
| Immediate | Active SMT | 0.06 | 0.002, 0.24 |
| 2 wk | Active SMT | 0.04 | 0.001, 0.15 |
| 4 wk | Active SMT | 0.09 | 0.003, 0.34 |
| *‘CI’ = credible intervals; ‘wk’ = weeks; ‘SMT’ = spinal manipulative therapy.* | | | |

## **Figure S2.** Bayesian summary panel plot.

Posterior summaries from a mixed-effects logistic model predicting blinding success (binary outcome yes/no). Bars/points show posterior medians with 95% credibility intervals on the probability scale (inverse-logit of posterior draws from estimated marginal means). (A) Probability by group (averaged over timepoints). (B) Main effect of pain change (averaged over groups and time). (C) Pain by Group interaction. (D) Time course by group.

## **References S1**

Betancourt M. The convergence of Markov chain Monte Carlo methods: from the Metropolis method to Hamiltonian Monte Carlo. Annalen der Physik 2019;531: 1700214.

Bürkner P-C. brms: An R package for Bayesian multilevel models using Stan. Journal of statistical software 2017;80: 1-28.

Carpenter B, Gelman A, Hoffman MD, Lee D, Goodrich B, Betancourt M, Brubaker M, Guo J, Li P, Riddell A. Stan: A probabilistic programming language. Journal of statistical software 2017;76: 1-32.

Colagiuri B, Sharpe L, Scott A. The Blind Leading the Not-So-Blind: A Meta-Analysis of Blinding in Pharmacological Trials for Chronic Pain. J Pain 2019;20: 489-500.

Gelman A, Carlin JB, Stern HS, Rubin DB. Bayesian data analysis Chapman and Hall/CRC. 1995.

Gelman A and Hill J. Data analysis using regression and multilevel/hierarchical models Cambridge university press. 2007.

Gelman A, Jakulin A, Pittau MG, Su Y-S. A weakly informative default prior distribution for logistic and other regression models. 2008.

Gelman A, Simpson D, Betancourt M. The prior can often only be understood in the context of the likelihood. Entropy 2017;19: 555.

Lewandowski D, Kurowicka D, Joe H. Generating random correlation matrices based on vines and extended onion method. Journal of multivariate analysis 2009;100: 1989-2001.

Neal RM. MCMC using Hamiltonian dynamics. Handbook of markov chain monte carlo 2011;2: 2.

Spiegelhalter DJ, Abrams KR, Myles JP. Bayesian approaches to clinical trials and health-care evaluation John Wiley & Sons. 2004.

Vehtari A, Gelman A, Simpson D, Carpenter B, Bürkner P-C. Rank-normalization, folding, and localization: An improved R ̂ for assessing convergence of MCMC (with discussion). Bayesian analysis 2021;16: 667-718.
